# New aspects of TGF-β superfamily signaling in development and disease (2022 FASEB meeting review)

**DOI:** 10.12703/r/11-36

**Published:** 2022-12-15

**Authors:** Stuart J Newfeld, Michael B O’Connor

**Affiliations:** 1 School of Life Sciences, Arizona State University, Tempe, AZ 85287-4501, USA; 2 Department of Genetics Cell Biology and Development, University of Minnesota, Minneapolis, MN 55455, USA

**Keywords:** TGF-β, Receptors, Smads, Structures, Therapeutics

## Abstract

The 13th Federation of American Societies for Experimental Biology (FASEB) Summer Research Conference, “TGF-β superfamily signaling in development and disease” was convened at the Grand Hotel in Malahide, Ireland in July 2022. The Transforming Growth Factor-β (TGF-β) family of secreted proteins consists of agents of intercellular communication found in all multicellular animals. Attending the meeting was a diverse group of scholars with shared interests in understanding TGF-β signaling mechanisms, normal functions, and the diseases associated with misregulation and mutation. Despite intense study over the previous 35 years, new features of TGF-β activity continue to be discovered. This meeting report offers 21 investigator-provided summaries that illustrate the breadth of the thought-provoking presentations. An emerging theme of the meeting was the power of cross-disciplinary studies, such as one combining immunology, biochemistry, and structural biology, to unravel the secrets of parasitic TGF-β mimics. Please join us at the next meeting.

## Introduction

The Transforming Growth Factor-β (TGF-β) family of secreted factors consists of ancient and conserved agents of intercellular communication in multicellular organisms. The family is the largest set of signaling molecules in vertebrates. The family affects many aspects of development, physiology, homeostasis, and tissue repair via a multitude of downstream pathways. Despite intense study throughout the previous 35 years in both model organisms and humans, new functions and mechanisms for regulating TGF-β activity continue to be discovered across many areas of biology.

**Figure 1.**
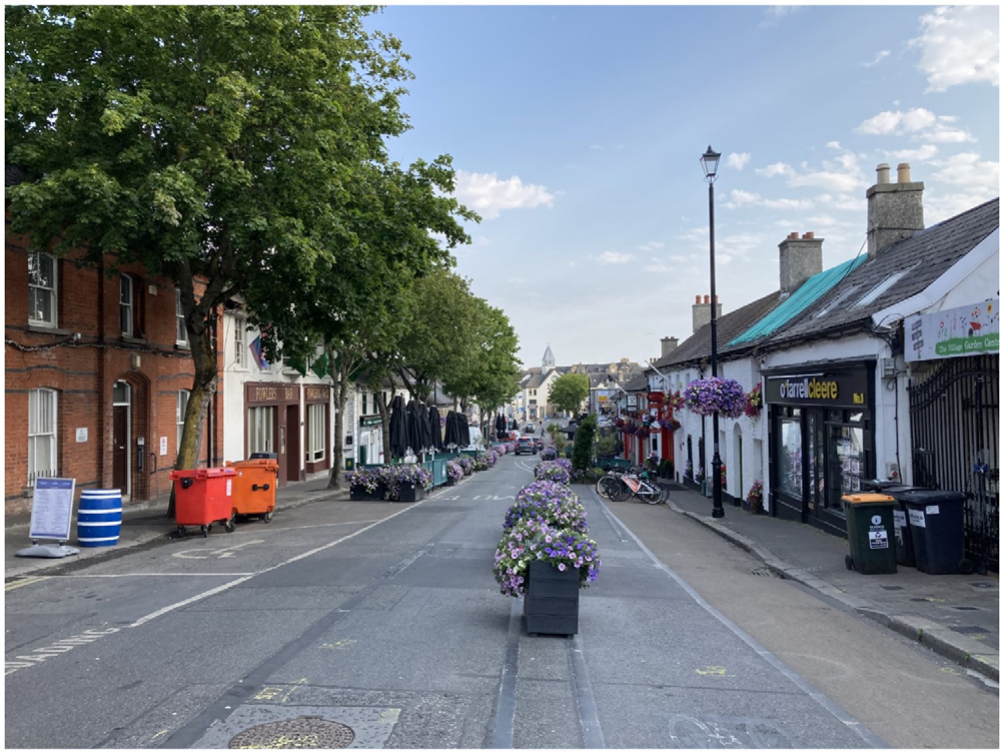
Downtown Malahide near the Grand Hotel.

**Figure 2.**
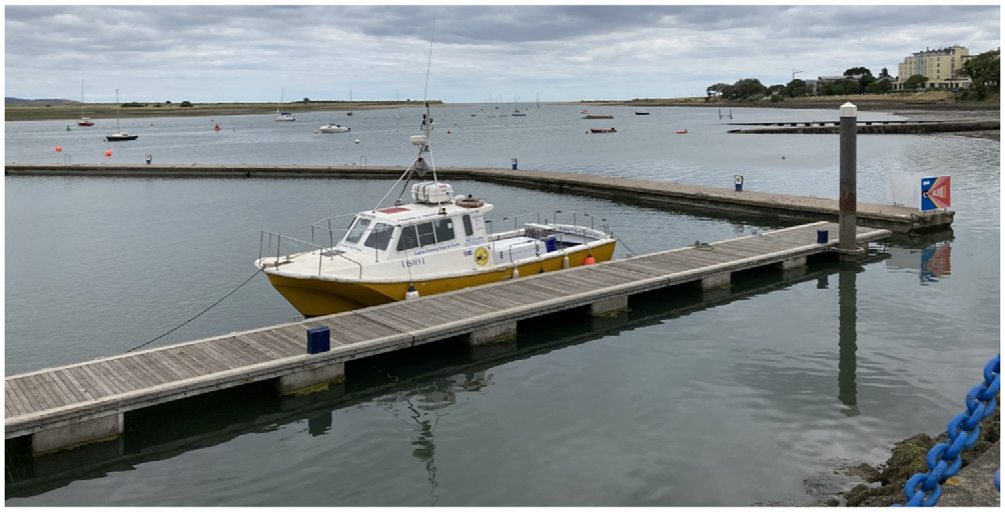
Malahide Harbor looking east toward the Irish Sea with the Grand Hotel on the right.

A recent Federation of American Societies for Experimental Biology (FASEB) Summer Research Conference at the Grand Hotel in Malahide, Ireland (see Figure 1 and Figure 2) brought together a diverse group of investigators with a shared interest in understanding the mechanisms of TGF-β signaling pathways and their normal developmental and homeostatic functions, together with the diseases engendered by pathway misregulation and dysfunction. This was the 13th meeting in a biannual series and was organized by Gareth Inman (Cancer Research UK & Beatson Institute, University of Glasgow) with co-organizers Cathy Savage-Dunn (Biology, Queens College, City University of New York) and Tom Thompson (Molecular Genetics, Biochemistry & Microbiology, University of Cincinnati). The goal was to discuss the latest discoveries in TGF-β pathway research, including emerging applications for therapeutics.

The TGF-β family is composed of three subfamilies: Activin, Bone Morphogenetic Protein (BMP), and TGF-β. In humans, for example, the Activin subfamily contains eight proteins, the BMP subfamily has 17, and the TGF-β subfamily has eight for a total of 33 family members. These ligands regulate a broad range of cellular processes (for a review, see 1). Regulatory mechanisms operate at many levels including transcription, secretion, processing in the extracellular matrix, via receptors, co-receptors, and a multitude of signal-transducing components. Regulation is further context-dependent according to cell type as well as temporal (developmental) and spatial (nearest neighbor) cues.

This report provides a sample of summaries of the thought-provoking presentations. Summaries were provided by the presenters and then edited for length, to reduce jargon and to add details for the non-specialist. The first author is responsible for any ambiguities introduced during this process. Half of the summaries reflect presentations by women. Presentations of younger scientists, including graduate students and postdoctoral fellows, were prioritized. First are summaries of immunology and disease, then development, followed by structure and signaling, and concluding with therapeutics.

### Immunology

Rick Maizels (Wellcome Centre Integrative Parasitology & Institute Infection, Immunity and Inflammation, University of Glasgow) presented intriguing results from studies of a parasitic worm that influences host TGF-β signaling through a family of 10 modular multi-domain proteins called TGF-β mimics (TGMs). TGMs are monomeric proteins that elicit the responses of TGF-β despite bearing no amino acid similarity. TGM-1 binds the TGF-β type I receptor TβRI (ALK5) and the TGF-β type II receptor TβRII through distinct domains. TGM-1 induces immune-suppressive regulatory T cells that allow the parasitic worm *Heligmosomoides polygyrus* to evade the immune system of its murine host. TGM-1 also binds to CD44 through a distinct domain.

Andrew Hinck (Structural Biology, University of Pittsburgh) followed with details from a recently reported structural study of TGM-1^2^. He noted that three Complement Control Protein (CCP)-like domains are employed to ligate TβRI with TβRII and initiate TGF-β signal transduction. Detailed analysis revealed that CCP-like domain 3 binds the same residues of TβRII as TGF-β while CCP-like domain 2 binds the same residues of TβRI as the TGF-β:TβRII complex. These domains bind to TβRI and TβRII with a 1:1:1 stoichiometry, as opposed to the 1:2:2 stoichiometry of the TGF-β heterotetrameric signaling complex. Overall, the experiments suggest that TGM family members have convergently evolved to closely mimic the function of TGF-β, though with variation among TGM proteins in their relative affinity for individual TGF-β receptors. Ongoing studies aim to identify the molecular basis for the abilities of different TGM proteins to target different cell types.

### Cancer biology

Kohei Miyazono (Graduate School of Medicine, University of Tokyo) discussed tissue-clearing technology that enables the comprehensive analysis of cancer progression. He described the application of tissue-clearing to a study of cancer metastasis^3^. He found that TGF-β both induced epithelial-mesenchymal transition (EMT) and suppressed the expression of E-cadherin in human lung adenocarcinoma A549 cells. Intravenous injection of TGF-β-treated cells into nude mice resulted in enhanced cell adhesion and survival of these cells at metastatic sites. There E-cadherin reappeared, possibly through a mesenchymal-epithelial transition. Furthermore, TGF-β-treated cells increased metastatic colonization of TGF-β-untreated cells when co-injected into mice. This data implies that TGF-β induces remodeling of the tumor microenvironment.

Mary Helen Barcellos-Hoff (Radiation Oncology & Comprehensive Cancer Center, University of California, San Francisco) updated us on the role of TGF-β signaling in promoting effective DNA repair^4^. Cells in which TGF-β signaling is lost or blocked rely on alternative end joining, an error-prone repair pathway that increases sensitivity to radiation and chemotherapy. She found that a gene expression signature for TGF-β signaling is anti-correlated with one for alternative end joining in 16/17 solid cancers in a tumor database. Those patients whose cancers are characterized as low TGF-β have a significantly better response to chemo/radiation therapy. Further exploration of the relationship between TGF-β signaling and the DNA damage response may identify new options for TGF-β inhibitors in cancer therapy.

Aristidis Moustakas (Medical Biochemistry & Microbiology, Uppsala University) discussed studies addressing two topics: the pro-tumorigenic, pro-proliferative action of TGF-β signaling and the regulation of extracellular vesicle (EV) biogenesis by TGF-β. First, he employed 3D mammosphere cultures to show that sustained TGF-β signaling supports robust proliferation and subsequent tumor initiation in recipient mice^5^. Second, he noted that mesenchymal breast cancer cells respond to TGF-β by EV secretion via mechanisms that involve vesicular trafficking. He then showed that the EVs can carry the TGF-β protein and the mRNA of several regulators of TGF-β signaling^6^.

Mythreye Karthikeyan (Pathology & Comprehensive Cancer Center, University of Alabama, Birmingham) shared data on the role of the Activin subfamily Inhibin proteins in ovarian cancer^7^. Inhibins are either homodimers or heterodimers comprising various combinations of Inhibin-α, Inhibin-βA (InhibinA), or Inhibin-βB (InhibinB) subunits. Inhibin-βA and Inhibin-βB homodimers often perform the opposite function of their respective heterodimers, earning them the nicknames ActivinA and ActivinB. In studies of tumors derived from ovarian cancer cell lines, she noted that hypoxia-induced tumor growth, angiogenesis, and vascular leakiness were dependent on elevated InhibinA levels. Functional studies demonstrated that InhibinA effects on ovarian cancer were mediated by the co-receptor endoglin and ACVRL1 (ALK1), a type I receptor shared by the Activin and BMP subfamilies.

### Bone and liver disease

Eileen Shore (Orthopedic Surgery, University of Pennsylvania) demonstrated that skeletal muscle regenerates poorly in *Acvr1^R206H^* knock-in mice. This genotype contains a missense mutation in the Activin type I receptor (ACVRI/ALK2) equivalent to one in humans that causes fibrodysplasia ossificans progressiva (FOP). This is a rare disorder characterized by heterotopic bone formation^8^. In FOP mice, muscle stem cells and a second type of stem cell that resides in muscle, fibro/adipogenic cells (these will differentiate into collagen-producing and fat-storing cells), proliferate normally after an injury. However, the fibro/adipogenic progenitors fail to decline normally during post-injury regeneration, leading to overgrowth. Also, during regeneration, muscle stem cells form underdeveloped muscle fibers that do not fuse into myotubes*.* The data identify a role for *ACVR1^R206H^* in inhibiting muscle regeneration in FOP.

Lopa Mishra (Bioelectronic Medicine, Feinstein Institutes for Medical Research) reported on her studies of mice heterozygous for mutations in both Smad3 and Sptbn1 (βII-spectrin). She had previously identified Smad3 as essential for the prevention of multiple liver pathologies^9^. In subsequent studies of *Aldh2^−/−^ Sptbn1^+/−^* (Aldh2 mutant and Sptbn heterozygous) mice that become obese on a normal diet, she observed that activated caspases cleave βII-spectrin and disrupt homeostatic Smad3 signaling. This disruption led to the activation of fibrogenic and oncogenic cascades in the liver. Treating Aldh2 mutant mice with a small interfering RNA (siRNA)-targeting βII-spectrin or crossing them into a background with a liver-specific knock-out of βII-spectrin reduced activation of fibrogenic and oncogenic cascades. The data indicated that βII-spectrin presents a means to target the Smad3 signaling pathway to modulate obesity.

Sabine Bailly (Health Biology & Biotechnology, University of Grenoble-Alpes) introduced data from her studies of liver homeostasis in BMP9 (GDF2) knock-out mice^10^. She showed that loss of BMP9 leads to spontaneous liver fibrosis via loss of endothelial fenestration (small dynamic pores that enable the passage of fluid and small solutes). She presented a comparative RNA sequencing (RNA-Seq) analysis of liver sinusoidal endothelial cells from BMP9 knock-outs compared with wild-type mice. The analysis identified over 2,000 differentially expressed genes. Gene Ontology analysis showed that the BMP9 knock-out led to a decrease in endothelial cell differentiation markers, reduced BMP and Notch signaling, and activation of the cell cycle. These observations support a central role for BMP9 in maintaining liver homeostasis.

### Developmental biology

Caroline Hill (Developmental Signaling, Francis Crick Institute) discussed her studies on endoderm progenitor specification in the zebrafish embryo. These cells form on the embryo side of the margin between the animal and vegetal poles. Endoderm cells appear in an apparently random “salt and pepper” pattern interspersed among mesoderm progenitors in response to a Nodal gradient^11^. She demonstrated that the Nodal gradient interacts with an overlapping Fibroblast Growth Factor (FGF) gradient to specify endoderm progenitors. Initially, the combined inputs of Nodal and FGF give rise to a population of bipotential cells in the first five cell rows adjacent to the margin. Subsequently, a subset of bipotential cells within the two rows adjacent to the margin stochastically switch to an endodermal fate, while the remainder becomes mesoderm. She noted that for bipotential cells, high levels of Nodal favored endodermal fate while high levels of FGF disfavored this fate.

Mayu Inaba (Cell Biology, University of Connecticut Health Center) shared studies of the BMP family member Decapentaplegic (Dpp) in the germline stem cell niche in *Drosophila*. Her data revealed a second function for Dpp in the testis germline^12^. It was already known that Dpp functions in germline stem cells to support the process of self-renewing mitosis. Employing genetically encoded nanobodies that block Dpp diffusion, she found that Dpp also functions in differentiating cells after they migrate away from the niche. The germline signal moves contact-dependently via nanotubes formed on stem cells, while the freely diffusing fraction of Dpp signals to cells outside the niche. The data showed that Dpp has the opposite effect on cells outside of the niche versus inside. Outside the niche, Dpp promotes cellular differentiation, whereas inside it stimulates mitosis.

Mihaela Serpe (Cellular Communication, National Institute of Child Health and Human Development) reported that *Drosophila* larval motor neurons accumulate the phosphorylated form of the BMP signal transducer Mad (pMad) not only in nuclei but also at the synapse. She proposed that synaptic pMad functions as a presynaptic sensor of postsynaptic activity. Consistent with this hypothesis, synaptic pMad remains anchored to presynaptic complexes containing BMP receptors that receive a retrograde BMP signal. Mutational analysis showed that a highly conserved H2 helix in Mad at the Mad-BMPRI interface modulates sensing activity^13^. She also noted that mutations in human Smad H2 helices had been seen in patients with neuronal deficits. She suggested that the H2 helix is critical for neuronal BMP function across species.

Hyungseok Kim, in the lab of Jan Christian (Neurobiology & Internal Medicine, University of Utah), presented new data on the regulation of Nodal signaling at the onset of gastrulation in *Xenopus* embryos^14^. He explained that the transmembrane protein Tril utilizes a two-pronged approach to regulate both arms of the TGF-β signaling cascade. Previous studies showed that Tril stimulates degradation of the inhibitory Smad7, thus enhancing BMP signals that induce hematopoietic fate in the mesoderm. New data revealed that Tril simultaneously dampens Nodal signaling by activating the ubiquitin ligase Nedd4L. He identified Pellino2 as a protein that connects Tril to Nedd4L and recruits Traf6 to this ubiquitin ligase complex to activate Nedd4L, which then targets Nodal receptors for degradation.

### Structural biology

Wei Li (Clinical Medicine, University of Cambridge) discussed the crystal structures of the type II receptor BMPRII in binary receptor complex with the ligand BMP10 and ternary signaling complex with the shared Activin/BMP subfamily type I receptor ALK1 and BMP10^15^. She showed that interactions between ALK1 and BMP10 are almost identical, whereas interactions between BMPRII and BMP10 are highly dynamic. Her structural data support the hypothesis that BMPRII-dependent responses require high concentrations of BMPRII to stabilize the signaling complex. As a consequence, *BMPR2* mutations that cause even modest haploinsufficiency will have the biggest impact on tissues requiring the highest levels of BMPRII, such as the lung. This helps explain why heterozygous *BMPR2* mutations result in pathology, such as pulmonary arterial hypertension.

Tom Thompson (Molecular Genetics, Biochemistry & Microbiology, University of Cincinnati) highlighted three recent advances in understanding how Activin subfamily signaling molecules bind and coordinate the assembly of their cognate receptors. First, he presented the structure of a complex containing two ligands ActivinA and GDF11 with an immunoglobulin heavy-chain fusion containing two Activin receptors: the type II ActRIIB and the type I ActRIA (ALK4)^16^. Second, he reported the structure of the TGF-β subfamily member anti-Müllerian hormone (AMH, or Müllerian-inhibiting substance [MIS]) in complex with its dedicated type II receptor AMHR2^17^. Third, he described the discovery that the unstudied ligand ActivinC signals through Smad2/3 via the Activin subfamily type I receptor ALK7^18^.

### Signaling mechanisms

Ying Zhang (Cellular & Molecular Biology, National Cancer Institute) reported data on the ability of Smad3 to act via a non-transcriptional mechanism^19^. The process is stimulated by the Epidermal Growth Factor (EGF)-induced phosphorylation of Smad3 at Thr179 in its linker region. This phosphorylation sends Smad3 away from transcriptional functions and toward forming complexes with Poly (rC)-Binding Protein 1. This complex cooperates with other splicing factors to regulate alternative splicing in cancer cells. One example is the switch to the exclusion of exon 12 from full-length Tak1. While the full-length Tak1 promotes apoptosis, the exon 12-excluded Tak1 stimulates TGF-β-induced EMT. Thus, the impact of Smad3 on Tak1 splicing turns a tumor-suppressing signal into a tumor-promoting one.

Peter ten Dijke (Cell & Chemical Biology and Cancer Genomics Center, Leiden University) communicated two studies employing a zebrafish model that describe the diverse roles that long non-coding RNAs (lncRNAs) play in TGF-β signaling. Note that lncRNAs do not become proteins and were only recently uncovered as regulators of the TGF-β family^20^. He reported that a previously unstudied lncRNA is induced by TGF-β signaling and functions as a signaling inhibitor to reduce cell migration and invasion. He balanced this with a report of another unstudied lncRNA induced by TGF-β signaling that has the opposite effect. The second lncRNA enhanced EMT, cell migration, and invasion. Overall, his conclusion is that lncRNAs are integral to not only the regulation but also the function of TGF-β pathways.

Michael Elowitz (Biology & Biological Engineering, HHMI, and California Institute of Technology) reported three aspects of BMP signaling. First, analysis of published single-cell RNAseq “cell atlas” data revealed that receptors are expressed in recurring combinations, termed pathway expression motifs^21^. These data are consistent with the hypothesis that distinct receptor combinations are receptive to distinct ligand combinations. Second, he presented experimental data showing how cells expressing different receptor combinations respond to different ligand combinations. From these data, ligands can be grouped into equivalence classes that exhibit similar responses and interactions with other ligands^22^. These equivalence groups are different for different cell types, such that ligands may signal equivalently in one context but non-equivalently in another. Third, he introduced a new *in vitro* system to reconstitute complex gradient-shaping behaviors such as ligand shuttling. These studies provide new insights into TGF-β signaling as a combinatorial spatio-temporal process.

Johannes Auth (Molecular Genetics, Weizmann Institute of Science), in the lab of Yaron Antebi^23^, addressed a related question: how do cells interpret simultaneous signals from TGF-β and BMP ligands? His single-cell RNA-Seq data was captured from cells with a pair of stably inserted reporter plasmids, each with either a BMP or a TGF-β response element. The analysis showed that the presence of both ligands decreased BMP and increased TGF-β responses across a wide range of ligand combinations. Computational modeling suggested that promiscuous Smad heteromerization was the underlying cause of the preference for TGF-β. He suggested that the benefit of this mechanism was to reduce ambiguity in signal transduction in the presence of a multi-ligand environment.

### Therapeutic applications

Aris Economides (Connective Tissue Diseases, Regeneron Pharmaceuticals) presented the unexpected outcome of studies with a selective antibody against ACVR1 in a mouse knock-in model of FOP (*Acvr1^R206H^*). The data showed that anti-ACVR1 antibodies that block signaling by the wild-type ACVR1 receptor instead activate signaling by the FOP mutant receptor ACVR1^R206H^^24^. This effect exacerbates rather than represses heterotopic ossification in the model. New information about the mechanism of action for the mutant receptor was obtained, but overall, the data indicate that this approach offers no benefit for patients with FOP. The silver lining to this cautionary tale is that the ability of anti-ACVR1 antibodies to block wild-type receptors has shown therapeutic benefit in a mouse model of trauma-induced heterotopic bone formation.

Rohan Manohar (Scholar Rock, Cambridge, MA) presented a new approach^25^ to reducing hyperactive TGF-β signaling in fibrotic kidney disease. He shared preclinical data for a selective antibody (Latent TGF-β Binding Protein [LTBP]-49247) that inhibits matrix-associated LTBP-TGF-β1 complexes. These disulfide-bonded complexes contain a monomer of LTBP and a dimer of TGF-β1. Therapeutically, LTBP-49247 reduced pSmad2 expression in a knock-out mouse model of Alport syndrome and reduced fibrosis in a rat model of chronic kidney disease. In these models, LTBP-TGF-β1 inhibition reduced circulating biomarkers of kidney damage, such as urea and creatinine. The therapeutic data, together with data from a dose-ranging 13-week preliminary safety study, suggests that LTBP-49247 may offer a better safety profile than other therapeutics for treating chronic kidney fibrosis.

## Summary

The TGF-β research community continues to advance our understanding of this multifaceted signaling pathway; new data was presented describing how it works, what it does, and what happens when it goes awry. Several of the talks broke new ground by applying state-of-the-art methods, such as single-cell RNA-Seq, to address fundamental questions. Others employed tried-and-true methods to characterize new features of the pathway, such as lncRNAs. The presence of talks on promising new therapeutics is always exciting. In the future, we expect more cross-disciplinary studies, such as the immunological, biochemical, and structural analysis of parasite TGF-β mimics. Please join the organizers Cathy Savage-Dunn and Tom Thompson, and co-organizer Mythreye Karthikeyan at the next meeting. Stay tuned for the dates and location.
